# Postural change for supine position does not disturb toddlers’ nap

**DOI:** 10.1038/s41598-020-68832-3

**Published:** 2020-07-20

**Authors:** Hidenobu Ohta, Yoshihisa Oishi, Takako Hirose, Sachiko Nakaya, Keiji Tsuchiya, Machiko Nakagawa, Hirotaka Gima, Isao Kusakawa, Hitoshi Yoda, Toshihiro Sato, Toru Sasaki, Hiroshi Nishida, Toshimasa Obonai

**Affiliations:** 1 Department of Pyschophysiology, National Institute of Mental Health, National Center of Neurology and Psychiatry, 4-1-1 Ogawa-higashi-cho, Kodaira, Tokyo 187-8553 Japan; 2 Department of Psychiatry, Asai Hospital, 38-1 Togane, Chiba, Japan; 3 Department of Neuropsychiatry, Akita University Graduate School of Medicine, Hondo 1-1-1, Akita, Akita 010-8543 Japan; 4 Department of Pediatrics, Japanese Red Cross Medical Center, 4-1-22 Hiroo, Shibuya-ku, Tokyo 150-8935 Japan; 5 Department of Pediatrics, St. Luke’s International Hospital, 9-1 Akashi-cho, Chuo-ku, Tokyo 104-8560 Japan; 6 Faculty of Regional Sciences, Tottori University, 4-101, Koyama-Minami, Tottori, 680-8550 Japan; 7 Department of Neonatology, Toho University Omori Medical Center, 6-11-1 Omori-nishi, Ota-ku, Tokyo 143-8541 Japan; 8 Department of Global Marketing, Unicharm Corporation, 3-5-27 Mita, Minato-ku, Tokyo 108-8575 Japan; 9 Kyo-Sei Shakai (Convival Society Lab), Unicharm Corporation, 1531-7 Wadahama Toyohama-cho, Kanon-ji, Kagawa 769-1602 Japan; 10 Department of Maternal and Neonatal Medicine, Tokyo Women’s Medical College, 8-1 Kawada-chou, Shinjuku-ku, Tokyo 162-0054 Japan; 11 Department of Pediatrics, Tama-Hokubu Medical Center, Tokyo Metropolitan Health and Medical Treatment Corporation, 1-7-1 Aoba-chou, Higashimurayama, Tokyo 189-8511 Japan

**Keywords:** Neonatology, Paediatric research

## Abstract

This study examined whether forced postural change from prone to supine during toddlers’ nap, a preventative measure taken in Japan for sudden unexplained death in childhood (SUDC), disturbs toddlers’ sleep. When the "Back to Sleep" campaign (BSC) was introduced to Japan in 1996, its recommendations were also applied to infants aged 1 year old and over with the expectation that the BSC recommendations may also contribute to a decrease in the occurrence rate of SUDC. Since then, Japanese nurseries have routinely conducted sleeping position checks and positional adjustments of toddlers every 5–10 min during naps. A total of 52 toddlers (age 18.4 ± 3.3 months, means ± SD) were continuously monitored for 8 h during daytime at nursery schools for wake-sleep status and body position (prone, supine and lateral) with actigraphs and 3-orthogonal-axis accelerometers. Out of the 52 toddlers, 24 toddlers adopted prone positions during naps, which were adjusted by nursery staff back to supine. When nursery staff manually changed the toddlers position from prone to supine, the toddlers either did not wake or woke only briefly (3.1 ± 4.9 min) and returned to sleep soon after the positional change. Our study indicates that manual change of toddlers’ sleeping position from prone to supine, a potential SUDC prevention method, does not disturb toddlers’ sleep during their naps.

## Introduction

The "Back to Sleep" campaign (BSC) by the American Academy of Pediatrics (AAP), which encourages carers to place infants in a supine position every sleep, has contributed to a decrease in the incidence of sudden unexpected death in infancy (SUDI), which applies to infants less than 1 year of age^1–3^ and includes such causes as sudden infant death syndrome (SIDS), suffocation and unidentified cause of death^4^. The AAP has also commented that the BSC may not be effective for reducing sudden unexplained death in childhood (SUDC), which applies to infants aged 1 year of age and older, since such infants can roll from prone to supine position themselves^1^. However, the exact mechanism that causes SUDI is still unknown and the AAP has admitted that there is a lack of scientifically solid data for determining the optimum age until when infants should be put to sleep in a prone position for SUDI prevention^1^. Recently, Crandall and Devinsky have emphasized the importance of epidemiologically evaluating the risk of SUDC, as well as the need for research into possible preventative measures for this category of deaths^5^. They also summarized that SUDC most frequently occurs in boys aged 1–3 years who are born full term as singletons (63–64%), and that victims are usually found unresponsive in the prone position (75%) with their faces down (50%) mostly during sleep (> 95%) in the winter (> 40%)^5–7^. During the SUDC risk period of 1–3 years of age, major changes to sleep distribution and duration rapidly occur in young children and a biphasic pattern of wake and sleep appears^8–19^.

When the BSC was introduced to Japan in 1996, unlike in the US, its recommendations were also applied to infants aged 1 year old and over with the expectation that the BSC recommendations may also contribute to a decrease in the occurrence rate of SUDC^20^. Since then, Japanese nurseries have routinely conducted sleeping position checks and postural adjustments of infants every 5–10 min during naps to avoid critical events related to SUDC. At the same time, however, nursery staff are generally concerned about the possibility of disturbing toddlers’ naps and causing their arousal when changing their nap position for SUDC prevention. In the current study, we observed the wake-sleep status and body positions of 52 toddlers between 1 and 2 years of age using actigraphy and accelerometers respectively to examine whether body positional change during naps disturbs their sleep.

## Results

**Figure 1:**
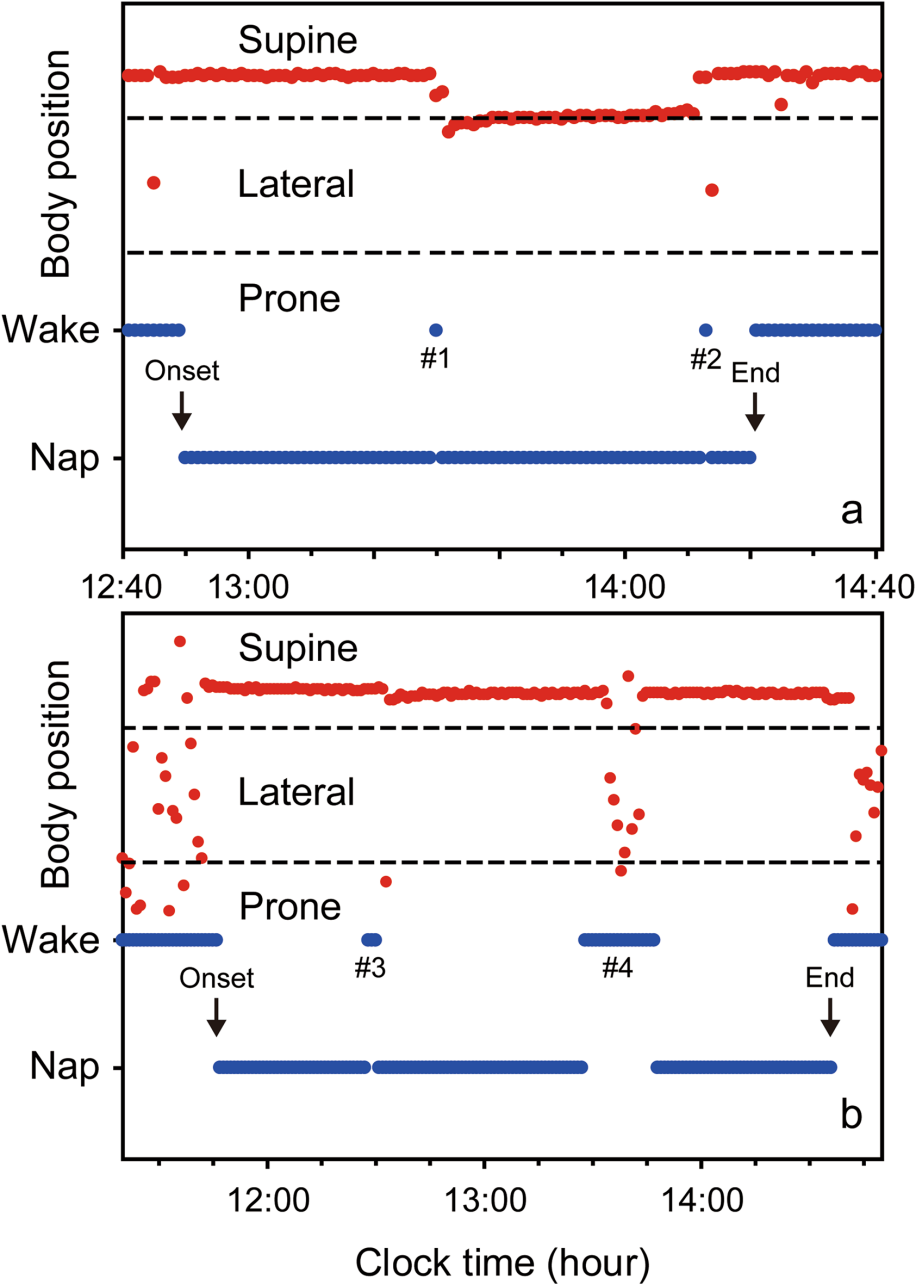
Representative daily activity-rest patterns of toddlers between 1 and 2 years of age during naps. The arrows indicate nap onset and end time, respectively. (**a**) After the toddler had transient wake #1 during nap, she had small body positioning changes within the supine position range and soon returned to sleep. After she had transient wake #2, she changed her body position from supine to lateral but soon returned to supine herself and went back to sleep smoothly. (**b**) After the toddler had transient wake #3 during nap, he changed his body position from supine to prone, leading to a forced postural change from prone to supine by a nursery staff member. Please note that he returned to sleep without any wake bout after the postural change. When he had another transient wake (wake #4), he again changed his body position from supine to prone and a nursery staff member returned his body position from prone to supine. However, he again changed his body position from supine to lateral and finally returned to supine himself and went back to sleep smoothly.

A total of 52 toddlers (age at recording: 18.4 ± 3.3 months, mean ± SD; 27 boys and 25 girls) were monitored to assess their naps at nursery schools during daytime for wake-sleep status and body position (prone, supine and lateral) with actigraphs and accelerometers that we had developed for a previous study of ours^21^. Toddlers’ nap environment is shown in Table 1. Figure 1 indicates two representative nap patterns for the activity and rest of toddlers between 1 and 2 years of age, demonstrating that various nap patterns exist among toddlers corresponding to their postural change during nap. All toddlers started their naps in supine position. Nursery staff changed the toddlers’ sleep positions to supine position only when the toddlers were found sleeping in prone position. Nursery staff did not change the toddlers’ sleep positions if the toddlers were sleeping in any non-prone position: either supine or lateral position. Out of the 52 toddlers, 24 toddlers returned their position from prone position to supine position with the assistance of nursery staff at least once during sleep, while 28 toddlers maintained a supine or lateral position until the end of their naps. Toddlers’ nap variables, such as nap-onset time, nap duration, nap-end time, nap sleep efficiency, WANO (wake after nap onset), and wake after postural change, are also indicated in Table 2. No statistical differences were found between the boys and the girls among any of the nap variables (*t* test, *p* > 0.05).

**Table 1:** Daytime nap environment.

	Number of responses (%)	
	Boys (n = 27)	Girls (n = 25)
Nursery nap environment		
In beds		
Yes	0(0)	0(0)
No	27(100)	25(100)
On floor with mattresses		
Yes	27(100)	25(100)
No	0(0)	0(0)
Regular breathing checks		
Yes	27(100)	25(100)
No	0(0)	0(0)
Nap during weeks		
Yes	26(100)	22(100)
No	0(0)	0(0)

**Table 2:** Daytime nap variables by sex (mean ± SD).

Nap variables	Total (n = 52)	Boys (n = 27)	Girls (n = 25)	P-value
Nap onset time	12:05 ± 0:27	11:59 ± 0:24	12:13 ± 0:29	0.0634
Sleep latency (min)	16.1 ± 6.4	17.3 ± 7.2	14.9 ± 5.2	0.1884
Nap duration (h)	2.2 ± 0.6	2.3 ± 0.6	2.2 ± 0.5	0.4532
Nap end time	14:18 ± 0:27	14:15 ± 0:29	14:22 ± 0:25	0.3595
Sleep efficiency (%)	93.3 ± 7.8	92.5 ± 8.9	94.1 ± 6.4	0.4680
WANO (wake after nap onset) (min)	9.5 ± 11.0	11.0 ± 12.8	7.8 ± 8.7	0.3006
Wake after postural change (bouts)	0.2 ± 0.4	0.1 ± 0.5	0.2 ± 0.4	0.4620
Wake after postural change (min)	3.1 ± 4.9	1.7 ± 1.2	3.8 ± 6.0	0.5684

Table 3 also demonstrates the differences in nap variables between the infants who were manually forced to change from prone to supine position (n = 24) and the infants in non-prone position (n = 28). As expected, infants who adopted a prone position during their naps experienced a more significant number postural-change-induced wake bouts since more postural changes were counted in these infants (*p* = 0.0001, *t* test, Table 3). However, no statistical difference was detected in wake durations after forced postural change by nursery staff during nap between the two groups (*p* = 0.0989, *t* test, Table 3), suggesting that forced postural change of the infants in prone position did not significantly cause them to experience more wake than the non-handled infants. Moreover, the infants who were manually changed from prone to supine position during nap had significantly longer nap durations than the non-handled infants, who maintained a non-prone position (*p* = 0.0208, *t* test, Table 3). In addition, the timing of the first wake of the infants who were manually changed from prone to supine position during nap was significantly later (i.e., longer WANO) than that of the non-handled infants, who maintained a non-prone position (*p* = 0.0232, *t* test, Table 3).

**Table 3:** Daytime nap variables by position (mean ± SD).

Nap variables	Total (n = 52)	Prone (n = 24)	None-prone(n = 28)	*P* value
Nap onset time	12:05 ± 0:27	11:57 ± 0:18	12:12 ± 0:32	0.0524
Sleep latency (min)	16.1 ± 6.4	16.1 ± 7.2	16.1 ± 5.7	0.9921
Nap duration (h)	2.2 ± 0.6	2.4 ± 0.6	2.0 ± 0.5	0.0208*
Nap end time	14:18 ± 0:27	14:21 ± 0:30	14:15 ± 0:24	0.4050
Sleep efficiency (%)	93.3 ± 7.8	91.3 ± 7.7	95.0 ± 7.5	0.0903
WANO(wake after nap onset) (min)	9.5 ± 11.0	13.2 ± 12.2	6.3 ± 9.0	0.0232*
Wake after postural change (bouts)	0.2 ± 0.4	0.3 ± 0.6	0.0 ± 0.2	0.0001**
Wake after postural change (min)	3.1 ± 4.9	3.7 ± 5.5	1.0 ± 0.0	0.0989

* < 0.05, ** < 0.001.

## Discussion

The present study produced two significant findings concerning the relationship between toddler’s postural change and their naps. First, the proportion of toddlers found to be in a prone position at least once during nap was a relatively high 46% (24 out of the 52 toddlers), indicating that toddlers between 1 and 2 years of age can change their body position from supine to prone, a possible risk factor for SUDC^5^. Regular sleeping position checks every 5–10 min during naps might contribute to reducing the time toddlers spend in a prone position. The body position accelerometer and actigraph also revealed that 22 of the 24 toddlers who adopted a prone position during nap only did so after they had a transient wake period during their nap, suggesting that transient wakes during their naps led to prone positions. Incidentally, of those 24 toddlers who adopted a prone position during nap, 2 cases had been in prone position for five minutes or more before being manually noticed by nursery staff. In contrast, the body position accelerometer could detect all incidences of toddlers sleeping in prone positions during naps within just one second, making monitoring by accelerometer considerably more advantageous than manual observation.

The second significant finding in this study was that forced body postural change of toddlers from prone to supine position mostly did not disturb their naps (nap duration: 2.2 ± 0.6 h). Out of the 24 toddlers detected in prone position, forced postural change from prone to supine positions led to no arousal in 15 toddlers while the remaining 9 toddlers experienced a brief period of arousal (3.1 ± 4.9 min) (Table 2). We also compared the difference in wake duration between the non-handled toddlers and the posture-adjusted toddlers after posture adjustments had been made. The result showed that no significant differences in wake durations after postural change during naps were detected between the two groups, indicating that toddlers’ nap is not disturbed by postural change from prone to supine (*p* = 0.0989, *t* test, Table 3). Through interviews with the nursery staff in this study, we have confirmed an anecdotal belief that positional change from prone to supine during nap induces toddlers’ wake. The present results, however, indicate that forced body postural change of the toddlers from prone to supine position contributed to keeping the toddlers in their supine positions without disturbing their naps and may be useful for SUDC prevention.

The incidence of SUDI, which applies to infants less than 1 year of age, is four times greater than the incidence of SUDC, which applies to infants aged 1 year of age and older^5^, leading to more public interest in SUDI prevention than SUDC prevention. Recently, however, the importance of epidemiologically evaluating the risk of SUDC has been emphasized since SUDC has still not been effectively eliminated from the child population and SUDC leads to 1.4 in every 1,000,000 toddlers aged 1–4 years dying each year in the US^5^. The parents of these toddlers also face great grief from not knowing the exact cause of their children’s death and are often investigated by law enforcement for possible crimes. Since children who die from SUDC are often found unresponsive in the prone position (75%)^5^, making toddlers maintain a supine position during nap would seem to reduce the risk of SUDC. The present study, which demonstrates no significant disturbance on child sleep from forced postural change from prone to supine during naps, will assure preschool staff that postural change of toddlers during naps is a suitable method for reducing the possible risk of SUDC.

In the interpretation of the results of this study, there are limitations that need to be considered. First, due to the relatively small sample size employed by this study (n = 52), analysis on sex and postural differences in actigraph variables may not be able to be fully performed, although the study is estimated to have a minimally adequate sample size for such analysis, as determined by previous studies^8,22^. An additional study using a larger sample size would be able to confirm the accuracy of the present results of the sex and postural differences in actigraph variables. Second, analysis of the effect of the other 2 types of nap-starting positions (prone and lateral) on naps variables was not investigated in this study as this would require performance of an RCT study which would force children to adopt all three different nap starting positions. Such an RCT study could help to confirm the accuracy of our findings that postural change in daily naps does not significantly induce the arousals of toddlers between 1 and 2 years of age. However, it would be ethically unsound for children to deliberately put into a prone position which might increase their risk of SUDC. Third, the purpose of the present study is only to encourage preschool staff to have a more favorable attitude toward maintenance of a supine position for children during naps for possible SUDC prevention. Further investigations on the effect of postural change for possible SUDC prevention are still required.

## Methods

### Subjects

Young toddlers between 1 and 2 years of age were collected as subjects from Tsuduki nursery (Yokohama, Japan), Futaba nursery (Yokohama, Japan), Katsuta nursery (Yokohama, Japan), Yokohama Port Side nursery (Yokohama, Japan) and Yamamomo nursery of Asai hospital (Togane, Japan). In the toddler recruitment process, parents received an oral explanation with handouts from either the nursery school’s principal or other nursery staff. Data were collected during the spring months (March to May) in 2018, during which there was an average room temperature of 16.1 °C, humidity of 67% and illuminance of a range of approximately 300–600 lx. Criteria for subjects for inclusion in the study were: (1) the absence of infection either current or within the past one week in the toddlers (2) lack of any mental/physical disorders in the toddlers. Criteria for exclusion was parental communication difficulties. Of the 54 toddlers originally deemed eligible, 2 were removed from the study because sleep data were corrupt due to technical trouble with the activity recording devices or insufficient recording of the daytime nap diary by nursery staff. As such, the resulting final sample was made up of 52 toddlers (27 boys, 25 girls). Sample size was determined using information from previous reports that measured the same sleep variables in toddlers with actigraphs^8,22^. We estimated that a minimum sample of approximately 20 toddlers would be sufficient to perform statistical comparisons of these sleep variables between two groups. The ethics committees at the Tama-Hokubu Medical Center and Asai Hospital approved the study protocol (No. T28-21, No. A2017042501) and all procedures were performed in accordance to their approved guidelines. Written informed consent was provided by the parents.

### Assessment of activity and sleep

#### Actigraphy

Actigraphy was employed for measurement of wake-time activity and sleep movement. This was performed by attaching a miniature wristwatch-like accelerometer to the wrist, ankle or waist of the subjects which continuously recorded movement over an extended period. The actigraphy device we employed in the present study was the Actigraph (Micro-mini RC, Ambulatory Monitoring Inc., NY, USA). Nursery staff were instructed to attach the Actigraphs to each child’s waist using an adjustable elastic belt for a period lasting approximately from 9:00 to 17:00 each day (weekdays). Waist attachment was considered to be the best method of attachment as it was found to be less disturbing than ankle or wrist attachment and has been reported to be an equally reliable data collection point as either the ankle or wrist^19,21^. The validity of sleep assessment by actigraphy has been confirmed using polysomnography in previous papers^23^.

Sampling of motility levels was performed in the zero-crossing mode in 1-min epochs. Actigraph resolution was set at 0.01 G/s. Activity data recorded on the Actigraphs was downloaded from the devices using ACTme software (ver. 3.10.0.3, Ambulatory Monitoring Inc., NY, USA). Sleep measurements were then analyzed using Action-W software (ver. 2.4.20, Ambulatory Monitoring Inc., NY, USA). Any intervals during the study when devices had to be removed for bathing etc. were recorded by the preschool staff in the sleep diary.

#### Sleep positions

The toddlers’ positions were monitored by an ADXL345 MEMS accelerometer (Analog Devices, MA, USA), with which acceleration of the body can be measured along 3 orthogonal axes^21^. All toddlers started their naps in supine position. Nursery staff returned the toddlers’ sleep positions to supine positon only when the toddlers were sleeping in prone position. Nursery staff did not change the toddlers’ sleep positions if the toddlers were sleeping in any non-prone positon: either supine or lateral position.

## Statistical analysis

Statistical analyses were carried out with SPSS Statistics 21.0 (IBM Corp. Armonk, NY, USA). Summary measurements are shown as means ± s.d.s. The sex and postural differences in sleep variables were analyzed using a t-test. Sample size calculations were based on an effect size of 0.6, with alpha = 0.05 and power = 0.90 using G*power^24^. A resulting sample size of n = 17 was found to be necessary to detect a difference in daytime nap variables between two independent groups. The sample size of n > 20 in each independent group in the present study fulfilled this sample size requirement.
